# Nanoparticle-boosted silymarin: Are we overlooking taxifolin and other key components?

**DOI:** 10.3389/fpls.2025.1628672

**Published:** 2025-08-15

**Authors:** Michal Selc, Radka Macova, Andrea Babelova

**Affiliations:** ^1^ Centre for Advanced Material Application, Slovak Academy of Sciences, Bratislava, Slovakia; ^2^ Department of Nanobiology, Cancer Research Institute, Biomedical Research Center, Slovak Academy of Sciences, Bratislava, Slovakia; ^3^ Department of Genetics, Faculty of Natural Sciences, Comenius University Bratislava, Bratislava, Slovakia

**Keywords:** nanoparticles, silymarin, silybin, flavonoid, taxifolin, elicitation

## Introduction

Nanoparticle-based elicitation represents an emerging technical advance in plant biotechnology that enables selective modulation of secondary metabolite pathways and induces mild stress, mainly due to its ability to influence plant growth, increase nutrient content, and improve both photosynthetic activity and metabolic processes (Wang et al., 2023b). The application of nanoparticles, such as TiO_2_, Fe_3_O_4_, or Ag-NPs, has been shown to be an effective way to stimulate the biosynthetic pathways of flavonoids, alkaloids, or phenolic acids in various medicinal plants (Gohari et al., 2020; Salih et al., 2022; Ahmed et al., 2023; Muhammad et al., 2025). As a result, they represent a promising tool for sustainable and economically advantageous production of plant extracts, which is important for phytotherapeutics with high demands on quality and consistency of composition.

This opinion highlights the need for a broader evaluation of nanoparticle-mediated enhancements in Silybum marianum beyond silybin, particularly focusing on the overlooked compound taxifolin and other flavonolignans. We seek to stimulate discussion on whether the observed therapeutic effects may result from a more complex phytochemical shift than currently appreciated, given that the biological activity of silymarin is influenced not only by silybin, but also by other constituents such as silychristin, isosilybin, silydianin, and taxifolin, some of which have demonstrated even stronger pharmacological effects in specific contexts.

## Changes in silymarin composition after nanoparticle treatment

Zinc oxide nanoparticles (ZnO-NPs) are attracting increasing attention for their potential to enhance the production of specialized plant metabolites (García-López et al., 2018; Wang et al., 2023a). In a recent study, Fahad Almulhim et al. (2025) investigated the use of ZnO-NPs to enhance silybin accumulation in *Silybum marianum* fruits and evaluated the osteoprotective potential of the resulting extracts (Fahad Almulhim et al., 2025). The foliar application of ZnO-NPs led to an almost eightfold rise in silybin (A+B) content compared to untreated controls, as determined by HPLC. The results are consistent with the previous work Jafari et al. (2023), who monitored the increase in silybins after treatment with titanium dioxide nanoparticles (TiO_2_-NPs). Foliar application of TiO_2_-NPs and chitosan significantly increased the accumulation of silybin A and B in milk thistle seeds. This effect was associated with the downregulation of specific microRNAs, leading to increased expression of key biosynthetic genes (Jafari et al., 2023). This highlights that nanoparticle treatment actively modulates regulatory pathways involved in secondary metabolism. This outcome points to a practical way of enhancing phytochemical yields without the need for major genetic engineering or intensive breeding efforts.

**Figure 1:**
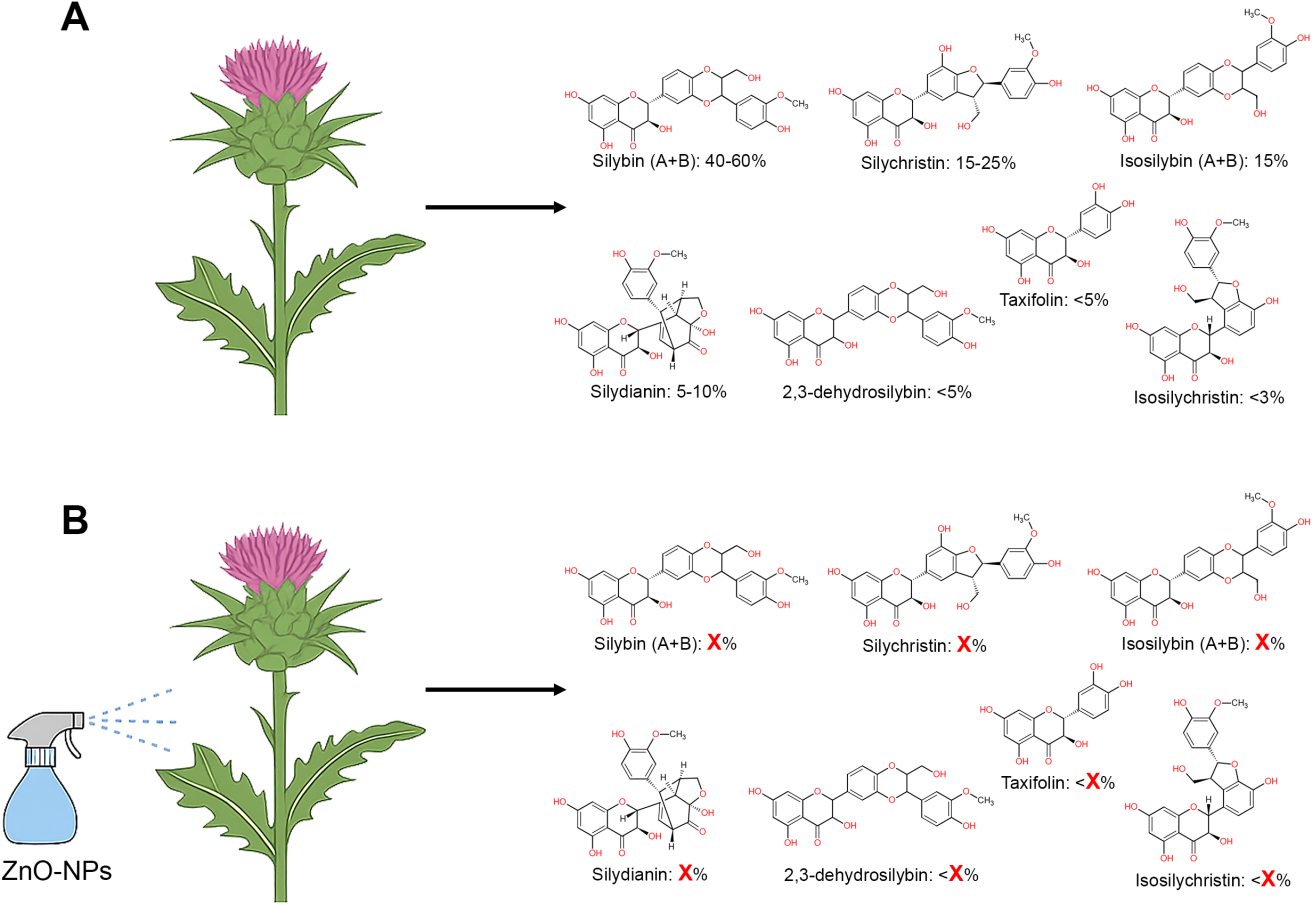
**(A)** Major components of silymarin, including their quantitative abundance. Silymarin consists of approximately 40-60% silybins, 15-25% silychristin, 15% isosilybins, 5-10% silydianin, less than 5% 2,3-dehydrosilybin or taxifolin, and less than 3% isosilychristin (Fenclova et al., 2019). **(B)** Hypothetical representation showing the unknown effect of nanoparticle treatment on individual flavonolignan and flavonoid content, emphasizing the need for detailed phytochemical profiling.

In both of the studies mentioned above, the presence of silybins was monitored. Silybin is considered as the key active compound of silymarin, an extract from the seeds of milk thistle, valued for its liver-protective, anticancer, antioxidant, and bone-preserving properties (Ray et al., 2024). Approximately 40-60% of silymarin is silybin, consisting of two isomers (silybin A and silybin B in a 1:1 ratio). Furthermore, silymarin contains other major flavonolignans such as silychristin, isosilybin A, isosilybin B, silydianin, 2,3-dehydrosilybin, isosilychristin, and the flavonoid taxifolin (**Figure 1A**). Understanding the broader shifts in silymarin composition after nanoparticle exposure is crucial not only for optimizing extract yield but also for ensuring reproducible and effective therapeutic outcomes.

## Discussion

Although the observed increase in silybin is promising, it raises some questions about the broader metabolic impacts of ZnO-NPs or TiO_2_-NPs treatment. Specifically, what exactly increased in the phytochemical profile after nanoparticle stimulation? (**Figure 1B**).

There are two main possibilities to consider:

1. Uniform enhancement of all flavonoids/flavonolignans. Nanoparticles treatment boosted the production of all flavonoids and flavonolignans across the board, representing a significant advantage. More bioactive compounds per biomass unit would mean smaller cultivation areas, reduced production costs, and more sustainable extraction process.
2. Selective enhancement or alteration of flavonoid/flavonolignan profiles. Nanoparticles may have selectively stimulated particular branches of the flavonoid biosynthetic pathway. This could have shifted the balance between different flavonoids and flavonolignans, creating an extract with evermore potent properties than the natural extract and potentially changing the biological properties of the extract.

Unfortunately, without a detailed comparison to extracts from untreated plants, it remains uncertain whether the profile of flavonoids/flavonolignans remained consistent.

Recent studies have shown that ZnO-NPs treatment leads to non-uniform changes in flavonoid synthesis. Wang et al. (2023a) reported that ZnO-NPs treatment in *Ginkgo biloba* caused an increase in the levels of kaempferol and isorhamnetin, while the content of quercetin remained largely unchanged (Wang et al., 2023a). This non-uniform shift suggests that nanoparticle treatment may not simply boost all flavonoid production equally. Similarly, other types of foliar spraying, including salicylic acid, spermine, and brassinosteroid, have revealed that the percentage changes across individual flavonolignans and flavonoid in silymarin are highly non-uniform. Some treatments led to a several-fold increase in one compound while simultaneously decreasing others, and vice versa in other cases, suggesting a highly selective and potentially competitive regulation within the silymarin biosynthetic pathway (Fanai et al., 2024). This supports the idea that nanoparticle treatment can induce localized or selective metabolic responses rather than a uniform boost in secondary metabolite content throughout the plant. These differences in flavonoid content are critical, as the biological activity of silymarin extracts depends not only on the amount of silybin but also on the presence and ratios of other flavonolignans (e.g., silychristin, isosilybin, silydianin) and flavonoid like taxifolin, which possess better medical properties than silybin. For example, isosilybin A and isosilybin B have shown superior and selective anticancer properties compared to silybin, particularly in hepatic and prostate cancer cell models (Deep et al., 2007; Polyak et al., 2010). Similarly, 2,3-dehydrosilybin and silychristin have demonstrated stronger antioxidant effects than silybin, especially in reducing intracellular ROS levels under oxidative stress (Jurčacková et al., 2025). Taxifolin and silydianin have also demonstrated more potent antioxidant and hepatoprotective activities than silybin, further supporting the idea that multiple constituents of silymarin may contribute significantly to its therapeutic profile (Anthony and Saleh, 2013; Kim et al., 2024).

Importantly, after the treatment with ZnO-NPs, or TiO_2_-NPs, increased expression of chalcone synthase (CHS), a key enzyme responsible for flavonoid biosynthesis pathway, was observed in milk thistle (Jafari et al., 2023; Fahad Almulhim et al., 2025). This upregulation is consistent with the general pattern of stress-related gene expression observed under nanoparticle exposure, likely mediated by ROS signaling and hormonal modulation. However, increased CHS expression alone does not indicate which downstream flavonoids or flavonolignans are preferentially synthetized, likely mediated by ROS signaling and hormonal modulation (Tripathi et al., 2022). In *Silybum marianum*, three CHS genes (*SmCHS1*, *SmCHS2*, and *SmCHS3*) with different expression patterns have been identified. *SmCHS1* and *SmCHS3* are highly expressed in petals during the early flowering stage and in stems and upper leaves at mid-flowering, and are most likely responsible for silymarin biosynthesis (Sanjari et al., 2015). *SmCHS2* is weakly expressed across plant tissues. This is consistent with the observation that ZnO-NPs treatment the most significantly increased *Sm*CHS3 expression, followed by *Sm*CHS1 and lastly *Sm*CHS2 (Fahad Almulhim et al., 2025).

Nevertheless, whether higher expression of CHS led predominantly to increased silybin, taxifolin, or a general rise in flavonoid flux remains unknown. The potential increase in taxifolin or other flavonoids would be noteworthy. Taxifolin is a flavonoid precursor in silymarin biosynthesis (AbouZid et al., 2017) with well-documented anti-osteoporotic effects. Satué et al. (2013) show that taxifolin promotes osteoblast differentiation and inhibits osteoclastogenesis, contributing to bone formation and preservation (Satué et al., 2013). These findings have been further confirmed by later studies, demonstrating that taxifolin promotes osteogenic differentiation of human bone marrow mesenchymal stem cells by enhancing the expression of osteogenic markers and inhibiting TNF-α-induced NF-κB signaling (Wang et al., 2017), and inhibits RANKL-induced osteoclastogenesis by modulating NF-κB signaling pathways (Zhang et al., 2019). Therefore, if ZnO-NPs treatment disproportionately upregulated taxifolin levels, the observed osteoprotective effects in the rat model might be partly or predominantly due to taxifolin rather than silybin (Fahad Almulhim et al., 2025).

Without a comprehensive phytochemical profile comparing nanoparticles-treated and untreated plant extracts, it is challenging to conclude which compounds are responsible for enhanced biological activity. However, what is certain is that monitoring only silybin levels is insufficient. Future studies should thus profile the full range of major flavonoids and flavonolignans and assess their relative abundances before and after nanoparticle treatment. In conclusion, published studies have made a valuable contribution to the field by demonstrating the potential of nanoparticles to enhance secondary metabolite accumulation in plants with therapeutical potential. Nevertheless, further investigations including more complex view are needed. Future research should focus on the follow:

1. Comparison of full flavonoid and flavonolignan profiles between treated and untreated plants.
2. Determination of disproportionality of taxifolin and/or other flavonoids increment.
3. Clarification of the observed therapeutical effects origin – its derivation from silybin or other components.

These findings represent an important step forward, and we hope that the insights presented here will guide future research. Our comments are intended to complement important findings and to support further advances in nanoparticle-assisted phytochemical enhancement.
